# CRISPR/Cas12a‐Enabled Multiplex Biosensing Strategy Via an Affordable and Visual Nylon Membrane Readout

**DOI:** 10.1002/advs.202204689

**Published:** 2022-11-28

**Authors:** Tao Hu, Xinxin Ke, Wei Li, Yu Lin, Ajuan Liang, Yangjing Ou, Chuanxia Chen

**Affiliations:** ^1^ The Children's Hospital Zhejiang University School of Medicine National Clinical Research Center for Child Health Hangzhou Zhejiang 310052 China; ^2^ International Peace Maternity & Child Health Hospital Shanghai Municipal Key Clinical Specialty Institute of Embryo‐Fetal Original Adult Disease School of Medicine Shanghai Jiao Tong University Shanghai 200030 China; ^3^ Center of Reproductive Medicine Shanghai First Maternity and Infant Hospital Tongji University School of Medicine Shanghai 201204 China; ^4^ School of Materials Science and Engineering University of Jinan Jinan Shandong 250022 China

**Keywords:** CRISPR‐Cas12a, multiplex nucleic acids detection, on‐site diagnosis, reverse dot blot, SARS‐CoV‐2

## Abstract

Most multiplex nucleic acids detection methods require numerous reagents and high‐priced instruments. The emerging clustered regularly interspaced short palindromic repeats (CRISPR)/Cas has been regarded as a promising point‐of‐care (POC) strategy for nucleic acids detection. However, how to achieve CRISPR/Cas multiplex biosensing remains a challenge. Here, an affordable means termed CRISPR‐RDB (CRISPR‐based reverse dot blot) for multiplex target detection in parallel, which possesses the advantages of high sensitivity and specificity, cost‐effectiveness, instrument‐free, ease to use, and visualization is reported. CRISPR‐RDB integrates the trans‐cleavage activity of CRISPR‐Cas12a with a commercial RDB technique. It utilizes different Cas12a‐crRNA complexes to separately identify multiple targets in one sample and converts targeted information into colorimetric signals on a piece of accessible nylon membrane that attaches corresponding specific‐oligonucleotide probes. It has demonstrated that the versatility of CRISPR‐RDB by constructing a four‐channel system to simultaneously detect influenza A, influenza B, respiratory syncytial virus, and SARS‐CoV‐2. With a simple modification of crRNAs, the CRISPR‐RDB can be modified to detect human papillomavirus, saving two‐thirds of the time compared to a commercial PCR‐RDB kit. Further, a user‐friendly microchip system for convenient use, as well as a smartphone app for signal interpretation, is engineered. CRISPR‐RDB represents a desirable option for multiplexed biosensing and on‐site diagnosis.

## Graphical abstract

CRISPR‐RDB platform represents a multiplex biosensor that could identify four targets in a single sample and convert target information into colorimetric signals on a piece of accessible nylon membrane. This sensor is sensitive and selective, cost‐effectiveness, instrument‐free, and visualization, and allows future integration into a microchip.

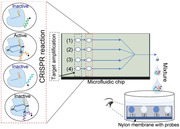

## 1 Introduction

Enabling multiplex nucleic acids detection in a single sample exhibits the advantages of robust diagnostic capacity, time‐saving, large information content, and higher confidence results than singleplex detection.^[^ ^1^, ^2^, ^3^, ^4^, ^5^ ^]^ Recently, the ongoing COVID‐19 not only presents similar clinical symptoms to other pathogens but also hinders accurate disease diagnosis in the existing co‐infection cases.^[^ ^6^, ^7^, ^8^, ^9^, ^10^ ^]^ To provide more definitive information, it is crucial to simultaneously distinguish the types of pathogens for controlling the source of infection and supportive treatments. The availability of multiplex‐enabled nucleic acids testing generally depends on quantitative polymerase chain reaction, microarrays, and next‐generation sequencing.^[^ ^11^, ^12^, ^13^ ^]^ However, these aforementioned methods require burdensome and costly manners that may not be available in laboratories with limited resources. Notably, the advent of point‐of‐care (POC) biosensor has sparked tremendous attention in clinical diagnostics owing to the merits of convenience, cost‐effectiveness, and time efficiency, and expands versatile strategies to achieve multiplex detection in miniaturized equipment.^[^ ^14^, ^15^, ^16^ ^]^

A promising alternative POC tool is the clustered regularly interspaced short palindromic repeats (CRISPR)‐associated (Cas) technology.^[^ ^17^, ^18^, ^19^, ^20^ ^]^ The origin of CRISPR/Cas technology was used for gene editing but has recently been repurposed in molecular diagnosis applications due to the finding of trans‐cleavage of any available non‐specific single‐stranded DNA (Cas12) or RNA (Cas13).^[^ ^21^, ^22^, ^23^, ^24^, ^25^ ^]^ It is an exciting technology with simple, fast, and robust performance. However, realizing CRISPR/Cas‐based multiplex biosensing is still challenging because of the indiscriminate collateral cleavage of unrelated probes. Some efforts have been made to address this issue. One attempt is the development of suitable Cas effector mixtures for unique cutting profiles. For instance, by employing heterologous Cas12a and Cas13a for trans‐cleavage of their corresponding probes, dual‐gene detection in a single tube was achieved.^[^ ^26^ ^]^ In addition, a multiplex and portable nucleic acids detection platform has been developed based on the cleavage preferences of LwaCas13a, CcaCas13b, LbaCas13a, and PsmCas13b for AU, UC, AC, and GA bases, respectively.^[^ ^27^ ^]^ This is an interesting attempt, nevertheless, still has the issues of signal crossing and leakage, as well as specialized fluorescent reader requirement. Another easier strategy is to use a multi‐channel design by manipulating spatial separation, which may be a realistic and effective solution that avoids interference with the advantages of CRISPR/Cas. For example, the combination of CARMEN (combinatorial arrayed reactions for multiplexed evaluation of nucleic acids) and Cas13 platform allows large‐scale detection using self‐organizing and miniaturized microfluidic technology.^[^ ^28^ ^]^ An electrochemical microfluidic biosensor in which the immobilization areas were separated into subsections to realize parallel detection of multiple targets has been designed.^[^ ^29^ ^]^ Nevertheless, this strategy is subject to several disadvantages of high‐cost instruments, skilled personnel, and sophisticated pretreatment processes, limiting POC and cost‐effective detection.

Although researchers have intelligently designed some methods to achieve multiplex biosensing, there is still huge room for converting these elegant ideas into commercial applications and on‐site diagnosis. A typical technique called reverse dot blot (RDB) enables efficient simultaneous detection of several mutations or different species in a single assay.^[^ ^30^, ^31^ ^]^ This technique generally involves the combination of PCR to amplify and label the regions of the DNA. The biotin‐labeled PCR products are subsequently employed as probes for hybridization with a nylon membrane that contains multiple specific‐oligonucleotide probes. After the addition of streptavidin‐horseradish peroxidase (HRP), the HRP is immobilized on the nylon membrane and efficiently catalyzes its substrate, 3,3″,5,5″‐tetramethylbenzidine (TMB), producing corresponding blue dots that could be directly recognized by the naked eye.

More significantly, this PCR‐based technique has been successfully developed into commercial diagnostic kits in the fields of prenatal diagnosis, virus genotyping, and newborn genetic disease screening.^[^ ^32^, ^33^, ^34^, ^35^ ^]^ To facilitate CRISPR/Cas multiplex biosensing, the RDB can be considered as an alternative signal readout tool by integrating CRISPR/Cas. This inspired us to establish a CRISPR/Cas12‐based RDB strategy for multiple target detection in which the activated Cas12a effector directly digests biotin‐labeled single‐strand DNA.

**Figure 1:**
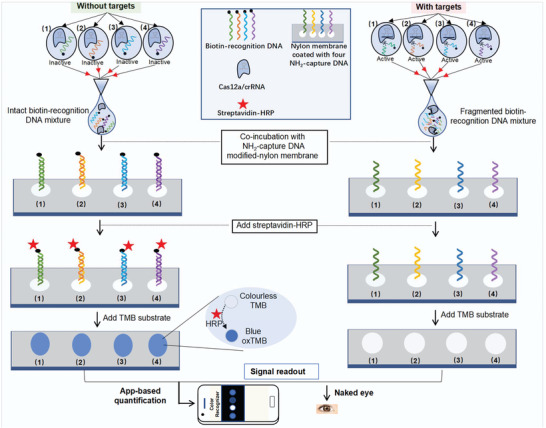
Schematic illustration of CRISPR‐RDB for multiplex nucleic acids detection. In the absence of targets, Cas12a is inactive, and the intact biotin‐recognition DNA could interact with its corresponding capture DNA on the surface of nylon membrane. After the addition of streptavidin‐HPR, stripe shows blue dots; In the presence of targets, the activated Cas12a cuts biotin‐recognition DNA into fragmented products. The strip without coating streptavidin‐HPR appears no blue dots.

Herein, a new solution, which we termed CRISPR‐based reverse dot blot (CRISPR‐RDB), has been proposed for multiplex nucleic acids detection at one test. We designed a strategy for the simultaneous detection of four targets as a model for proof of concept. The working mechanism is illustrated in **Figure** **1**. First, four pairs of recognition DNA (recDNA) and capture DNA (capDNA) were elaborately designed. The capDNAs were immobilized in parallel on a tiny nylon membrane. Each biotin‐labeled recDNA was mixed with one specific Cas12a‐crRNA, in which the crRNA was capable of identifying a specific target. In the absence of targets, the Cas12a effector is silenced and the biotin‐recDNAs are still intact. Subsequently, the as‐prepared nylon membrane was incubated with a mixture of the reaction solutions for the four channels. After the addition of streptavidin‐HRP, the colorless TMB substrate turns blue by efficiently catalyzing TMB substrate, presenting a color signal output that can be detected with a portable device or the naked eye. In the presence of targets, the activated Cas12a effector enables digesting of the biotin‐recDNA into fragments that cannot interact with the corresponding capDNA probe, and no signal is yielded. More significantly, the applicability of the CRISPR‐RDB platform was validated by testing influenza A virus (FA), influenza B virus (FB), respiratory syncytial virus (RSV), and SARS‐CoV‐2 (COV) clinical samples, exhibiting high application potential. In addition, the CRISPR‐RDB platform, with only a slight modification of crRNAs, was also constructed to test real human papillomavirus (HPV) samples, achieving high consistency and saving two‐thirds of the testing time in comparison to a commercial PCR‐RDB diagnosis kit.

## 2 Results and Discussion

### 2.1 Validation of CRISPR‐RDB on Multiplex Nucleic Acids Detection

**Figure 2:**
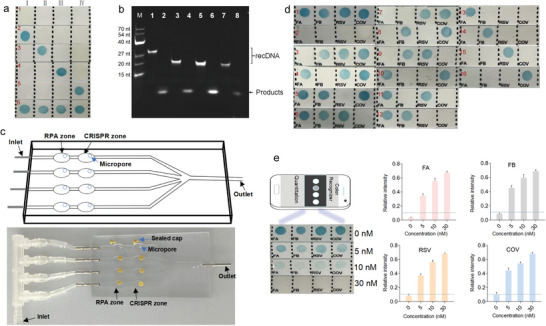
Validation of CRISPR‐RDB on multiplex nucleic acids detection. a) Strips showing results of the hybridization of matched recDNA and capDNA: strip 1, no recDNA; strips 2–5, incubation with recDNA I, recDNA II, recDNA III, and recDNA IV, respectively; and strip 6, incubation with recDNAs I, II, III, and IV. b) Denaturing PAGE characterization of trans‐cleavage of recDNA by activated Cas12a effector. Lanes 1, 3, 5, and 7 showing recDNAs I, II, III, and IV in the CRISPR reaction condition in the absence of targets, respectively; lanes 2, 4, 6, and 8 showing cut recDNAs I, II, III, and IV in the CRISPR reaction condition in the presence of targets (20 nM), respectively. CRISPR reaction condition: 100 nM Cas12a‐crRNA, 75 nM recDNA, and 50 nM crRNA incubated at 37 °C for 20 min. c) Schematic illustration of the four‐channel microfluidic chip (top) and photograph of an actual four‐channel microfluidic chip (bottom). d) Analysis of the feasibility of CRISPR‐RDB for multiplex nucleic acids detection in the absence of targets (strip 1) and in the presence of one kind of target (strips 3–6), two kinds of targets (strips 7–12), three kinds of targets (strips 13–16), or all four kinds of targets (strip 2). e) Color recognition of blue dots using a smartphone app in response to different concentrations of stDNA (0, 5, 10, 30 nM) (left); relative intensity quantitation for corresponding strips of 0, 5, 10, 30 nM (right).

In advance of establishing the CRISPR‐RDB platform for four‐channel nucleic acids detection, we first embarked on designing four pairs of single‐stranded DNAs including recognition DNA (recDNA I, II, III, and IV) and their corresponding capture DNA (capDNA I, II, III, and IV) (sequences listed in Table S1, Supporting Information). Polyacrylamide gel electrophoresis (PAGE) analysis revealed that all paired sequences displayed high specificity (Figure S1, Supporting Information). According to previous reports,^[^ ^31^ ^]^ the amino‐modified capDNA I, II, III, and IV were individually conjugated onto the surface of a negatively charged nylon film using 1‐ethyl‐3‐(3‐dimethylaminopropyl) carbodiimide strategy. Derived from **Figure** **2**a, recDNA I, II, III, and IV were all capable of bonding to their corresponding capDNA, leading to distinct blue dots from strips 2 to 5 as expected. Further, after the simultaneous injection of four recDNAs, four distinct blue dots appeared (strip 6).

The Cas12a effector exhibits robust trans‐cleavage of non‐target single‐stranded DNA upon target recognition.^[^ ^36^, ^37^ ^]^ It is of utmost importance to explore whether the designed recDNA could be cut by the activated Cas12a effector. We here employed FA, FB, RSV, and COV as model targets for proof‐of‐concept. We reasonably synthesized the crRNA sequences as well as the sense strands and antisense strand sequences for synthetic target DNAs (stDNAs, sequences listed in Table S1, Supporting Information). The PAGE analysis experiment was carried out to evaluate the performance of Cas12a effector in cleaving its corresponding recDNA strand. In contrast to bands 1, 3, 5, and 7, we found that the four kinds of recDNA were completely digested into short products (bands 2, 4, 6, and 8 in Figure 2b), which simultaneously contained Cas12a‐crRNAs and matched stDNAs (Figure 2b), indicating a high cleavage efficiency for the designed recDNAs.

Microfluidic chips have aroused strong interest owing to their potential as miniaturized devices that can integrate complicated sample‐handling steps, enable POC diagnosis, and empower multiplex analysis.^[^ ^38^, ^39^, ^40^, ^41^, ^42^, ^43^ ^]^ To facilitate operation, in this study, we introduced a hand‐held microfluidic chip allowing simultaneously testing of four targets. As illustrated in Figure 2c, the chip was composed of four‐parallel microfluidic channels, where each channel includes two zones: RPA zone allows RPA‐based amplification and CRISPR reaction zone allows Cas12a‐mediated recDNA cleavage. The chip bottom was designed as thin as possible (0.15 mm) to achieve a precise incubation temperature (37 °C) in the two zones (see Supporting Information, SA.1 and Figure S2 for detailed information) and a micropore was individually designed for the injection of RPA buffer and CRISPR buffer. In addition, a portable thermostatic incubator was likewise designed to equip the precise temperature in each step (Figure S2, Supporting Information). After sequential target amplification and CRISPR detection, the chamber solutions are collected together and involved in the subsequent hybridization assay.

To evaluate the feasibility of the proposed CRISPR‐RDB platform, the matched stDNA targets were directly injected into the CRISPR zone, and the collected reaction solutions were subsequently incubated with a piece of modified nylon film. As shown in Figure 2d, strip 2 emerged positive results by the naked eye in the presence of four kinds of targets, whereas strip 1 produced four blue dots without any targets. Strips 3–6 showed the results in presence of only one kind of target. More to the point, strips 7–12 and strips 13–16 randomly contained two and three kinds of targets, respectively. In brief, the results indicated that the CRISPR‐RDB platform is feasible for multiplex detection. In addition, the introduction of a smartphone device that possesses powerful data reception and processing capability enables quantitative analysis of blot dots by capturing red, green, and blue values (A detailed protocol for blue dots analysis is shown in SA.2 of Supporting Information). The relative intensity of each strip could be easily read by the smartphone in response to different concentrations of respective stDNA (left, Figure 2e). As shown in the right panel of Figure 2e, the relative intensity of blue dots presented a positive correlation to increasing concentrations of targets. We reasoned that the smartphone‐based readout exhibited a satisfactory performance for four‐channel nucleic acids detection.

### 2.2 Analytical Performance of CRISPR‐RDB

**Figure 3:**
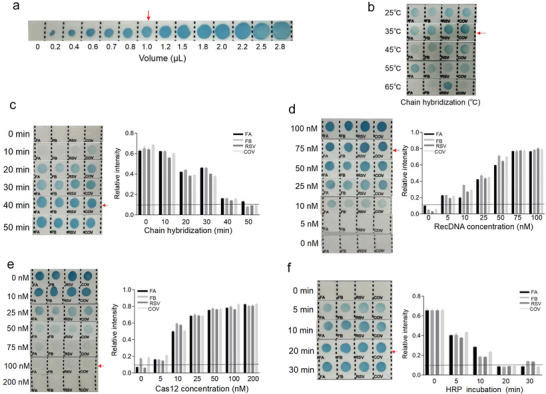
Optimization of critical parameters of CRISPR‐RDB platform. a) Optimization of the carrying volume on the nylon membrane (each square size is 0.5 cm × 0.6 cm in size). b) Evaluation of the effects of hybridization temperature between capDNA (1 µL, 10 µM) and recDNA (75 nM). c) Evaluation of the effects of hybridization time between capDNA ((1 µL, 10 µM) and recDNA (75 nM). d) Evaluation of the effects of recDNA concentration. e) Evaluation of the effects of Cas12a concentration on recDNA (75 nM). f) Evaluation of the incubation time with HRP. Red arrows show the optimal parameters.

Prior to using the CRISPR‐RDB platform for multiplex assays, we focused on tuning the output signal to make it more sensitive and robust. Derived from **Figure** **3**a, the optimal volume carried by the nylon membrane was 1.0 µL. As the hybridization conditions between capDNA and intDNA have a significant effect on the detection performance, the appropriate hybridization temperature and time were 35 °C (Figure 3b) and 40 min (Figure 3c), respectively. The concentration of recDNA plays a vital role during the hybridization event. It was found that the color intensity increased gradually with increasing recDNA concentrations, reaching a maximum value at 75 nM (Figure 3d). Based on the optimal recDNA concentration, it is of great importance to explore the well‐matched collateral activity of Cas12a. According to Figure 3e, the optimal Cas12a concentration in this study was 100 nM. The effect of streptavidin‐HRP incubation time was also investigated, and 20 min was found to be optimal (Figure 3f). A standard protocol has been proposed (shown in the experimental section) after the parameters optimization.

**Figure 4:**
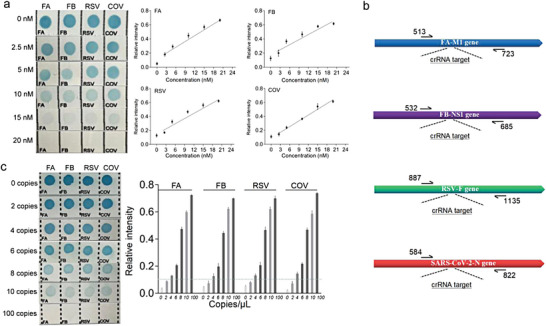
Analytical performance of CRISPR‐RDB. a) Strips results (left) and relative intensity quantitation (right) for different stDNA target concentrations (0, 2.5, 5, 10, 15, 20 nM). b) Schematic diagrams of the full‐length M1 gene of FA, NS1 gene of FB, F gene of RSV, and N gene of COV with crRNA target sites and RPA primers sites. c) Evaluation of the detection sensitivity of CRISPR‐RDB for real samples in combination with an RPA kit. Visual results (left) and relative intensity quantitation of FA, FA, RSV, and COV (right).

Next, we evaluated the performance of CRISPR‐RDB by simultaneously detecting four kinds of stDNA. As shown in **Figure** **4**a, the blue dots intensity in the four channels became gradually weak accordingly with the increase of respective stDNA concentrations. Relative quantification for a range of stDNA target concentrations using the smartphone app revealed good linearities in the range of 2.5–20 nM (Figure 4a, right). The limits of detection (LOD) were individually calculated to be 0.50, 0.90, 0.70, and 0.65 nM (3*σ*/*s*, in which *σ* is the standard deviation of the blank and *s* is the slope of the linear dynamic range). Furthermore, we conducted a study to demonstrate the lowest target concentration tested. As shown in Figure S3, Supporting Information, our assay can actually achieve the detection of targets below 1.5 nM for all four channels. To further assess the sensitivity of CRISPR‐RDB in real samples in addition to pseudovirus of COV, we implemented RPA to amplify the defined target nucleic acids with low concentrations. The crRNA and RPA primers sites from the specific M1 gene of FA,

NS1 gene of FB, F gene of RSV, and N gene of COV (Figure 4b, sequences listed in Table S1 and Table S2) were designed, where the performance of primers have been proved by comprehensive PAGE analysis of amplification bands (Figure S4). Obviously, the strips showed the gradient effect together with the concentrations of defined nucleic acids (0, 2, 4, 6, 8, 10, and 100 copies) (Figure 4c), suggesting the CRISPR‐RDB platform in combination with the RPA kit could detect as low as 4 copies per reaction in real samples.

**Figure 5:**
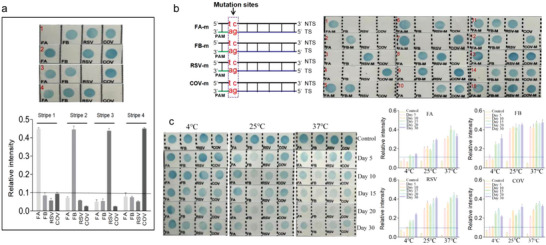
Analytical performance of CRISPR‐RDB. a) Evaluation of the mutual interference of the CRISPR‐RDB platform based on visual results (upper) and color intensity quantitation (lower). b) Schematic diagram of the two mutation sites (positions 1 and 2) in the stDNAs (left) and evaluation of the specificity of CRISPR‐RDB using the stDNAs with the two base mutations (right). c) Evaluation of long‐term storage of the nylon film probes at 4 °C, 25 °C, and 37 °C based on visual images (left) and color intensity quantitation (right).

Additionally, the four samples were analyzed separately to evaluate the specificity of the CRISPR‐RDB platform. Taking FA as an example, the assay clearly exhibited high selectivity for discriminating the target via crRNA‐DNA manner (strip 1, **Figure** **5**a), the same for FB, RSV, and COV (strips 2—4, Figure 5a). It is widely known that the discrimination of mismatched bases is particularly significant for the identification of disease‐related point mutations. We found that Cas12a effector is gradually silenced as the increased distance away from the protospacer adjacent motif (PAM) site when there are two or more mismatches in the crRNA‐target duplex using CRISPR‐RDB (Figure S5, Supporting Information).^[^ ^17^ ^]^ We next investigated whether the CRISPR‐RDB platform was capable of detecting multiple mutation sites in a single test. To this end, we employed two base‐pair mismatched targets (position 1—2, Figure 5b) that critically affect the trans‐cleavage activity of Cas12a effector (Figure S5, Supporting Information) for the proof of concept. We then

used random targets containing from one to four kinds of mutation targets. As seen in Figure 5b (right), the strips showed negative signals once the mutated targets were introduced into the assay. The results suggested that CRISPR‐RDB may provide a new approach for multiple mutation‐associated disease diagnosis.

To investigate platform stability, we mainly focused on the preservation condition of the labeled nylon film. As shown in Figure 5c (left), with the time prolongation, the performance of the prepared membrane was seriously affected after storage at 25 °C and 37 °C, probably because the single‐stranded DNA readily degrades at higher temperatures. Derived from Figure 5c (right) and Figure S6, Supporting Information, for further analysis, the optimal storage condition of membrane‐labeled probes does not exceed 10 days at 4°C and 3 days at 25 °C.

### 2.3 Evaluation of CRISPR‐RDB on Clinical Virus Samples

**Figure 6:**
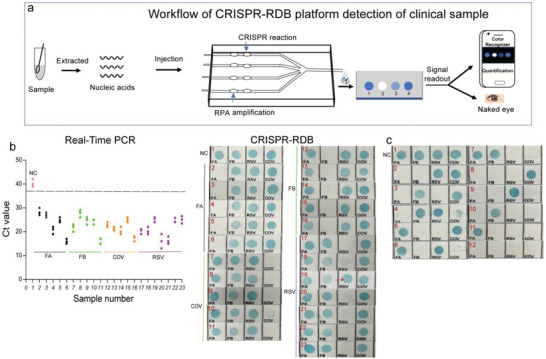
Evaluation of CRISPR‐RDB on clinical samples. a) Schematic diagram showing the workflow of the CRISPR‐RDB platform for four‐channel detection in clinical samples. The turnaround time: nucleic acids extraction (10 min), RPA amplification (15 min), CRISPR reaction, and hybridization (80 min), including signal readout. b) Results of real‐time PCR (left, three parallel runs for each sample) and CRISPR‐RDB (right, the red arrow indicates an inconsistent result) of 23 clinical samples (Nos. 1: negative control; Nos. 2–6: five positive samples of FA; Nos. 7–11: five positive samples of FB; Nos. 12–16: five positive samples of COV; Nos. 17–23: seven positive samples of RSV). c) Evaluation of the multichannel performance using samples containing random combinations of FA, FB, RSV, and COV (No. 1: negative control; Nos. 2–7: samples containing two viruses; Nos. 8–11: samples containing three viruses; Nos. 12: samples containing four viruses).

To illustrate the application of the CRISPR‐RDB in real samples, the workflow of the CRISPR‐RDB platform for clinical samples is presented in **Figure** **6**a, where the overall sample‐to‐answer turnaround time is approximately 105 min. Equal volumes of nucleic acid extracts were equally added into the RPA zone for rapid amplification. After CRISPR reaction, the four‐channel reaction solutions were collected for subsequent hybridization with probes on nylon film. The conspicuous detectable signal could be read by a smartphone app or by the naked eye. We further evaluated the usability of CRISPR‐RDB platform for the detection of FA, FB, RSV, and COV in clinical samples, including twenty‐two positive samples and one negative sample, tested by real‐time PCR (Figure 6b, left; Figures S7 and S8; Table S3, Supporting Information). As described in Figure 6b, the results obtained using CRISPR‐RDB were in agreement with the real‐time PCR results for all samples except one (strip 19). Furthermore, we verified the multiplex performance by preparing mixed virus samples. It can be derived from Figure 6c that CRISPR‐RDB exhibited high sensitivity and specificity for single or multiple targets, demonstrating its potential in the field of multiplex nucleic acids detection.

### 2.4 Application Advantages of CRISPR‐RDB

**Figure 7:**
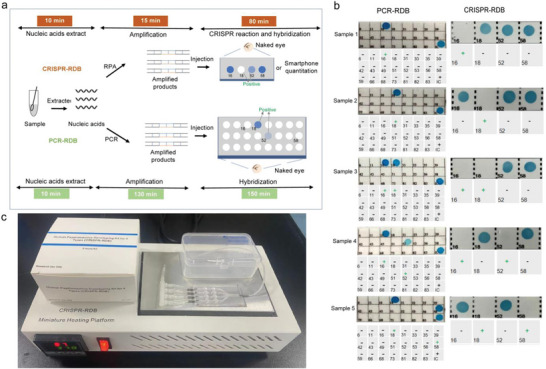
Application advantages of CRISPR‐RDB. a) Comparison of the detection workflow between PCR‐RDB and CRISPR‐RDB. b) Results of PCR‐RDB (“IC” indicates the control group) and CRISPR‐RDB for five positive samples (Sample 1: HPV of 16 type; Sample 2: HPV of 18 type; Sample 3: HPV of 16 and 18 types; Sample 4: HPV of 16 and 52 types; Sample 5: HPV of 18 and 58 types). c) Main laboratory equipment required for CRISPR‐RDB (Kit, microfluidic chip, miniature heating platform, and incubation box).

Cervical cancer, which is ascribable to persistent infection with high‐risk HPV, is the second most common malignancy in women around the globe.^[^ ^44^ ^—^ ^48^ ^]^ The predominant high‐risk HPV genotypes involved in invasive cervical cancer are HPV 16, 18, 31, 33, 35, 45, 52, and 58. At present, PCR‐RDB as a common diagnostic method has been widely used in the precise identification of HPV types. Based on the CRISPR‐RDB strategy, it is very simple and convenient to construct a four‐channel HPV diagnosis means by slightly altering the crRNA sequences. We then designed the crRNA and RPA primers (Figure S9 and Table S2, Supporting Information) for the frequent high‐pathogenicity types 16, 18, 52, and 58, and the HPV‐related CRISPR‐RDB platform could detect as few as five copies per reaction in real samples (Figure S10). Compared to PCR‐RDB (**Figure** **7**a), CRISPR‐RDB has a relatively fast turnaround time (105 min vs 290 min of PCR‐RDB), which is attributed to the introduction of isothermal amplification, powerful CRISPR technology, and proper design of hybrid sequences between recDNA and capDNA hybrid sequences. To validate its multiplex nucleic acids diagnostic performance, we preferentially tested five samples using a commercial PCR‐RDB kit (Figure 7b, left). The confirmed clinical samples were subsequently tested using the CRISPR‐RDB platform, achieving consistent results in agreement with the PCR‐RDB results for all five samples (Figure 7b, right). It highlights the commercial potential of the CRISPR‐RDB platform for HPV diagnosis with the advantages of simple operation, no need for expensive equipment, and time‐saving. More practically, Figure 7c shows the main equipment required for the CRISPR‐RDB assay, including a box of diagnostic kit (Figure S11, Supporting Information), CRISPR‐RDB miniature heating platform, handy chip, and a piece of nylon film with HPV probes.

## 3 Conclusion

In this study, we have developed a CRISPR‐RDB platform for the simultaneous detection of four nucleic acid targets by using a tiny and cheap nylon membrane for signal readout. The crRNA enables the recognition of the corresponding target; The handy microchip system enables the partition of four samples into a separate microfluidic chamber, each zone recognizes their target by well‐designed Cas12a‐crRNA from the same sample; The employed RDB technique enables the low‐cost, instrument‐free, and multiple signal output (see Supporting Information, SA.3 for detailed comparison with commercial methods). The applicability of the CRISPR‐RDB platform was confirmed by analysis of four kinds of virus samples by comparison with the gold standard qPCR, showing consistent results with clinical samples. More significantly, CRISPR‐RDB platform, with only a slight modification of crRNAs, was also evaluated to test HPV clinical samples, achieving high consistency and saving two‐thirds of testing time in comparison to a commercial PCR‐RDB method.

However, further improvement in signal output mode, pre‐amplification, and manual operation is needed to make it a truly “plug‐in” POC method for multiplex nucleic acids testing. Despite a lost signal manner, this assay displays good signal interpretation outcomes. In addition, a gained signal mode will be taken into consideration in our future work, where we will employ the toehold‐mediated strand displacement reaction (TSDR) through our group's previous works.^[^ ^44^ ^]^ Tyramine signal amplification (TSA), as an enzyme‐mediated signal amplification technique, has been applied to the detection of proteins and nucleic acids.^[^ ^49^ ^]^ It may provide a new clue to introduce multi‐HRP immobilized on the nylon membrane with the aid of TSA strategy, thus achieving ultra‐sensitivity detection instead of preamplification step. Recently, a one‐step CRISPR detection method (termed sPAMC) has been reported,^[^ ^50^ ^]^ where isothermal amplification and trans‐cleavage of Cas12a occur simultaneously in the same tube by use of suboptimal PAMs. It may provide us an idea that RPA amplification and recDNA cleavage could be achieved one reaction zone. In addition, it is necessary to develop a portable device by integrating an automatic microfluidic system together with the simple sample treatment to further shorten the sample‐to‐answer turnaround time. We ambitiously believe that CRISPR‐RDB platform may provide a simple, versatile, affordable, and efficient solution for multiplex nucleic acids detection in the future.

## 4 Experimental Section

### Materials

All the oligonucleotides used in the study were synthesized by General Biol (Anhui) Co., Ltd (Chuzhou, China) and the sequences were presented in Tables S1 and S2, Supporting Information. Nylon membrane (N66) was purchased from Cobetter Filtration Equipment Co., Ltd (Hangzhou, China). Sodium dodecyl sulfate (SDS), 3,3′,5,5′‐Tetramethylbenzidine (TMB), 1 × TE buffer, 20 × SSC buffer and HRP‐labeled streptavidin were obtained from Beyotime Biotechnology (Shanghai, China). 1‐Ethyl‐3‐(3‐dimethylaminopropyl) carbodiimide (EDC) were obtained from Sigma‐Aldrich, Inc. (St. Louis, MO, USA). Both RT‐RPA and RAA nucleic acids amplification kits were purchased from Jiangsu Qitian gene Biotechnology Co., Ltd. The TransScript Uni All‐in‐One First‐Strand cDNA Synthesis SuperMix was obtained from Transgen Biotech (Beijing, China) and the TB Green Fast qPCR Mix was purchased from Takara (Tokyo, Japan). LbCas12a (cpf1) was purchased from Guangzhou Magigen Biotechnology Co., Ltd. The pseudovirus of SARS‐CoV‐2 (11900ES) was obtained from Yeasen Biotechnology (Shanghai) Co., Ltd. Buffers used contained 20 × SSC (3 M NaCl, 0.3 M sodium citrate, pH = 7.0), MES buffer solution (50 mM MES, pH = 6.0), 5 × TBE buffer (90 mM Tris‐HCl/borate, 2 mM EDTA, pH = 8.0) and 1 × TE buffer (10 mM Tris‐HCl, 1 mM EDTA, pH = 8.0). Ultrapure water (Milli‐Q synthesis, Millipore Inc., Bedford, MA) was used throughout. All chemical reagents were of analytical grade.

The influenza virus A and B, respiratory syncytial virus samples were obtained from The Children's Hospital of Zhejiang University School of Medicine, which has been approved by the ethical committee of The Children's Hospital of Zhejiang University School of Medicine (2021‐IRB‐182). Human papillomavirus (HPV) samples were obtained from the international peace maternity & child health hospital (Shanghai), which has been approved by the ethical committee of MCHH on Human Research (ZH2018QNA37).

Preparation of nylon membrane: N66 was chosen as the support membrane and the label on the membrane was printed by a laser printer. The nylon membrane modification was prepared based on the previous report with a minor modification.^[^ ^32^, ^33^ ^]^ The N66 membrane was soaked by 5% EDC that was prepared in MES buffer solution for 30 min. Afterward, the N66 membrane was washed three times with ddH_2_O and was dried at 65 °C for 35 min. Subsequently, 10 µM NH_2_‐capture DNA dissolved in 4.2% NaHCO_3_ was added to the membrane. After placing at room temperature for 40 min, the membrane was blocked for 8 min with 100 mM NaOH. Finally, the membrane was washed three times and dried at 65 °C for 30 min. The membrane was stored at 4 °C for further use.

### RT‐qPCR

The RNA templates of Flu A, Flu B, RSV, and SARS‐CoV‐2 were extracted by RaPure Viral RNA/DNA kit and were converted into cDNA via TransScript Uni All‐in‐One First‐Strand cDNA Synthesis SuperMix for qPCR according to the manufacturer's instructions. qPCR was conducted using specific primers with the TB Green Fast qPCR Mix and was performed on StepOnePlus Real‐Time PCR System (Applied Biosystems, Foster City, CA, USA). The specificity of the primers was verified by melting curve analysis.

### PAGE Experiment

18% nondenaturing polyacrylamide gel electrophoresis was used to explore the annealing specificity of NH_2_‐capture DNA and biotin‐recognition DNA. Each well was loaded with prepared samples mixed with 6 × DNA loading buffer. The gel electrophoresis was performed at 120 V for 150 min using 0.5 × TBE buffer as the electrophoresis solution. The gels were imaged on Gel Doc XR (Bio‐Rad, Richmond, CA, USA).

Denaturing polyacrylamide gel electrophoresis was conducted to analyze the cleaved products of biotin‐recognition DNA. The whole 20 µL of reaction solution contained 1 µL of 10 µM FAM‐recognition DNA, 2 µL of 1 nM target, 1 µL of 2 µM Cas12a, and 2 µL of 1 µM crRNA. The mixture was incubated at 37 °C for 30 min. Then, 6 × DNA loading buffer was added into the solution and the mixture was loaded into a 20% denaturing polyacrylamide gel containing 8 M urea. The gel was visualized by Gel Doc XR (Bio‐Rad, Richmond, CA, USA).

### Assay of RPA Zone and CRISPR Zone

RT‐RPA and RPA assays were conducted based on the instructions of the commercial kit. The total 25 µL RT‐RPA reaction contained 1.0 µL of 10 µM forward and reverse primers, respectively; 12.5 µL reaction buffer V, 4.0 µL water, 5.0 µL template. The mixture was vortexed and spun shortly. Then, 1.5 µL of 280 mM MgOAc was added, and incubated at 37 °C for 15 min. The total 25 µL CRISPR reaction contained 100 nM Cas12a‐crRNA, 200 nM biotin‐recognition DNA, and was incubated at 37 °C for 20 min after the induction of amplification products.

### CISPR‐RDB Platform Detection Procedure

25 µL RT‐RPA or RPA buffer and 30 µL CRISPR reaction were injected into the RPA zone via micropore, respectively. Meanwhile, 20 µL nucleic acids extract was equally added into the RPA zone. The CRISPR‐RDB miniature heating platform was then set at 37 °C for 15 min. After that, a fraction of the reaction buffer of RPA zone into the CRISPR zone was carefully pushed. Gently shaking the chip and the chip was placed onto the heating platform for 20 min at 37°C. Then, the Cas12a effector was denatured by heating the mixture at least 60 °C for at least 5 min. After that, the overall buffer into a miniature container that contains a nylon membrane by the induction of a 5 mL hybridization solution (2 × SSC and 0.1% SDS) from the inlet of a five‐way valve (Figure S2b, Supporting Information) was pushed. After 40 min of incubation at 37 °C. Afterward, the nylon membrane was incubated with HRP‐labeled streptavidin (dilution of 1:15 000) for 15 min. The nylon membrane was thoroughly washed four times. Finally, the nylon membrane was incubated with TMB solution (1 mL) for 5 min and the readout could be measured by the naked eye and quantified by a smartphone app.

### Statistics Analysis

Each experiment was repeated three times. The results were presented as mean ± S.D. Correlations were performed with linear regression to determine the goodness of fit using Graphpad Prism 8.0.

## Conflict of Interest

The authors declare no conflict of interest.

## Supporting information

Supporting Information

## Data Availability

The data that support the findings of this study are available from the corresponding author upon reasonable request.
