# Supplementary material for: CRISPR/Cas12a‐Enabled Multiplex Biosensing Strategy Via an Affordable and Visual Nylon Membrane Readout
Source: Adv Sci (Weinh). 2022 Nov 28;10(2):2204689. doi: 10.1002/advs.202204689 (PMC9839848; doi:10.1002/advs.202204689)
Supplement: Supplementary file 1 — Supporting Information [file ADVS-10-2204689-s001.pdf]

ADVANCED SCIENCE
Open Access

Supporting Information

for *Adv. Sci.*, DOI 10.1002/advs.202204689

CRISPR/Cas12a-Enabled Multiplex Biosensing Strategy Via an Affordable and Visual Nylon Membrane Readout

Tao Hu, Xinxin Ke, Wei Li, Yu Lin, Ajuan Liang, Yangjing Ou and Chuanxia Chen**

Supporting Information

CRISPR/Cas12a-enabled multiplex biosensing strategy via an affordable and visual nylon membrane readout

Tao Hu, Xinxin Ke, Wei Li, Yu lin, Ajuan Liang, Yangjing Ou and Chuanxia Chen**

1. Section A: Results and Discussion

SA.1 Design of the microfluidic chip. The four-channel microfluidic chip using polycarbonate material was designed by AutoCAD software and processed by the numerical control carving machine. The overall dimensions of the chip are 55 mm (length) × 30 mm (width) x 4 mm (thickness). The maximum volume of each zone is about 28 μL. In this study, the chip was used as a carrier for RT-RPA/RPA and CRISPR reaction. Thus, when the chip is placed onto the self-fabricated CRISPR-RDB miniature heating platform, the chip bottom should be as thin as possible (0.15 mm, Figure S2c). In order to add the corresponding reaction liquid (RT-RPA/RPA buffer and CRISPR buffer) into their corresponding zones, we also designed a 1 mm circular hole in each zone (Figure 2c). Actually, this chip is not necessarily used in this study, but it is able to significantly improve the convenience of detection anywhere in combination with the CRISPR-RDB miniature heating platform. However, there is a huge room in terms of reducing manual steps, for example, by adding a micro-injection pump, the reaction is precisely injected into the reaction zone and the entire liquids are automatically extruded into a container containing a nylon film.

SA.2 Screenshots of the developed color recognizer application running on a smartphone.

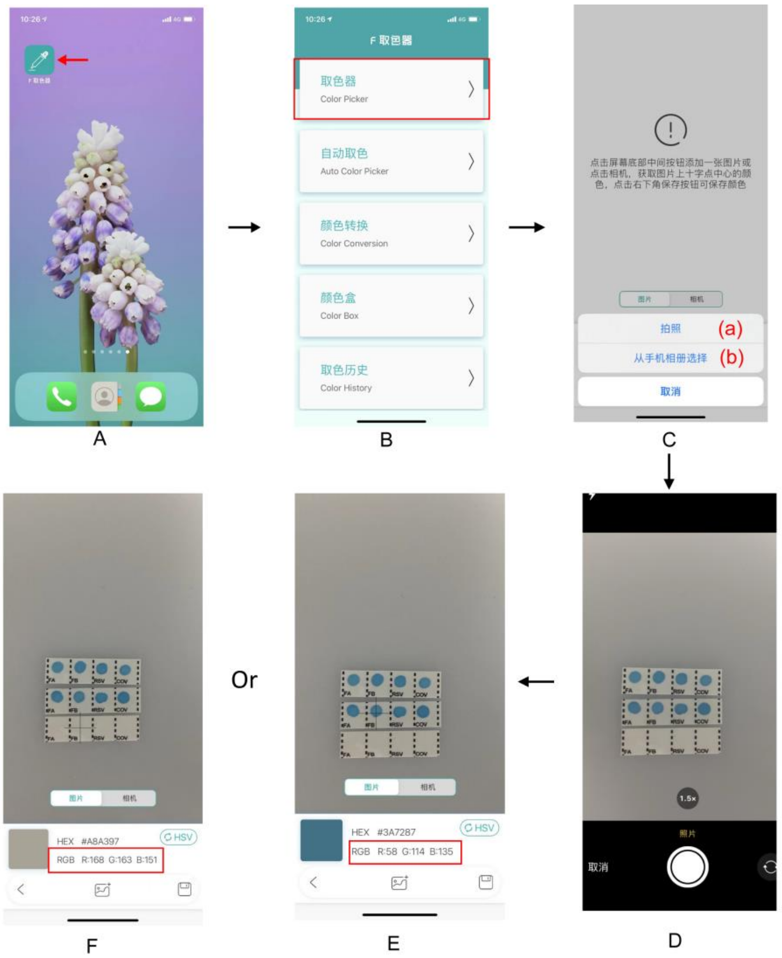

(A): Color recognizer application (APP) is shown. (B): APP runs and click "Color picker" button (red frame). Then, show the "Take picture (a)" or "Load picture (b)" button. (C, D): Alternatively, clicking the "Take picture" button to activate the smartphone camera and capture the images. (E, F): Move the black cross box to the blot area to acquire its corresponding RGB values (red frame). Then, the relative intensity value (Red/Green) was calculated and used as a parameter for quantitative analysis. Furthermore, we set a cut-off value (0.1, dash line in histograms) according to the deviation of 2 standard negative control values.

SA.3 Comparison of CRISPR-RDB platform with commercial products. As we all know, the quantitative polymerase chain reaction (qPCR), microarrays, next-generation sequencing, and PCR-reverse dot blot require expensive apparatus, professional lab, complicated preparation, and time-consuming. In this work, our proposed method only used an inexpensive self-made heater within 105 minutes to achieve results anywhere. In addition, the CRISPR-RDB platform using reagents and materials is available. In brief, this method has the advantages of cost-effectiveness, instrument-free, ease of preparation, and shows great potential in the field of multiple nucleic acids diagnosis in the future. We summarize the features of CRISPR-RDB versus commercial methods as follows.

Summary features of CRISPR-RDB vs commercial methods

**Methods**	**Detection time (hours)**	**Materials cost**	**Apparatus cost**	**Scale of targets**	**On-site diagnosis**
PCR-RDB	~5	Low	High	Medium	No
qPCR	~2.5	Low	High	Medium	No
Microarrays	~5	High	Very high	Large	No
Next-generation sequencing	~120	High	Very high	Large	No
This work	~1.7	Low	Very low	Small	Yes

2. Section B: Supporting Figures and Tables

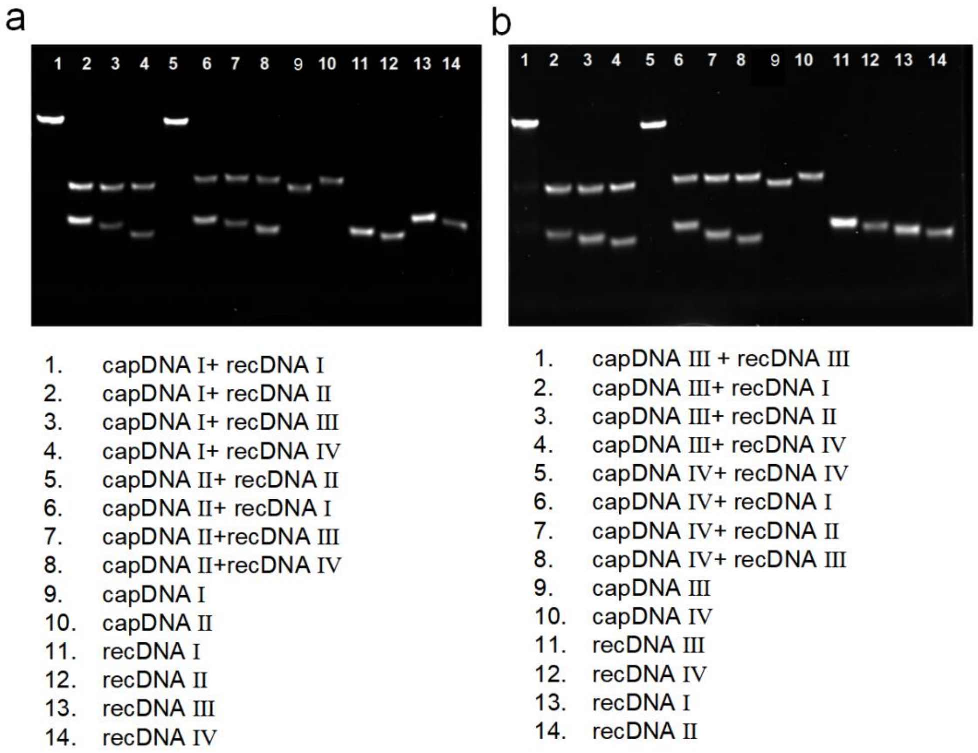

Figure S1. Native PAGE analysis of the specificity of the designed sequences between recDNA and capDNA. a) The specificity analysis of the pairs I and II. b) The specificity analysis of the pairs III and IV.

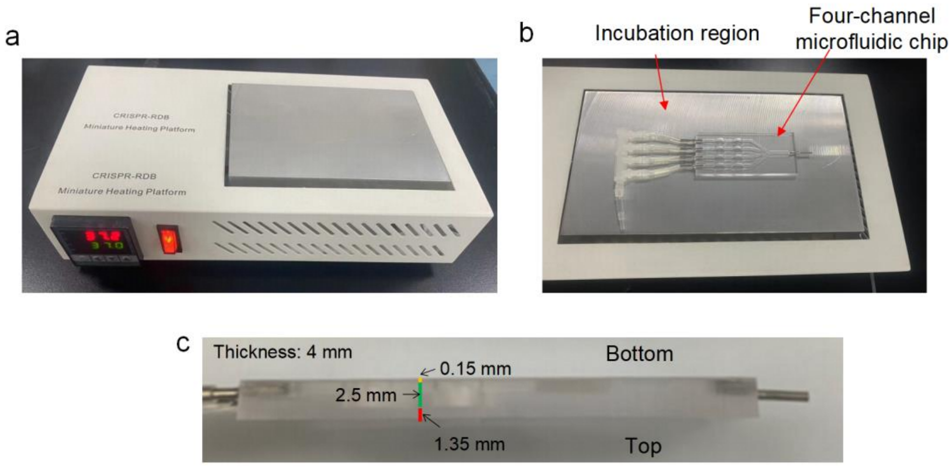

Figure S2. Self-fabricated CRISPR-RDB miniature heating platform (Providing an accurate temperature control for the microfluidic chip and hybridization reaction). a) Front view of the instrument. b) Front view of the four-channel microfluidic chip incubation on the surface of instrument. c) Side view of microfluidic chip. Thickness is 4 mm; The width of reaction zone is 2.5 mm; The distance from the bottom surface to the reaction zone is 0.15 mm; The distance from the top surface to the reaction zone is 1.35 mm.

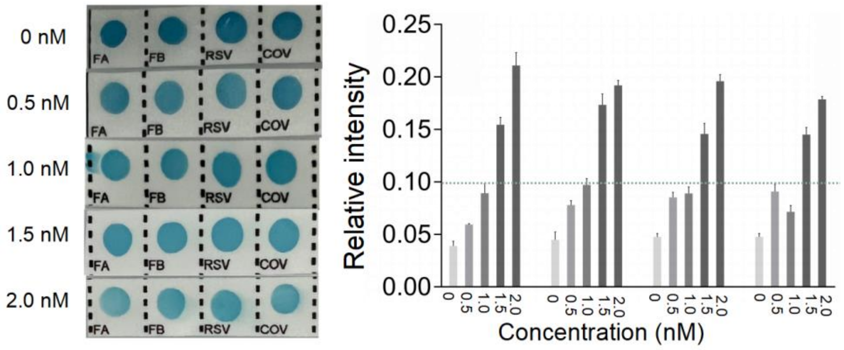

Figure S3. Strip results (left) and relative intensity quantitation (right) for the stDNA target concentrations (0, 0.5, 1.0, 1.5, and 2.0 nM).

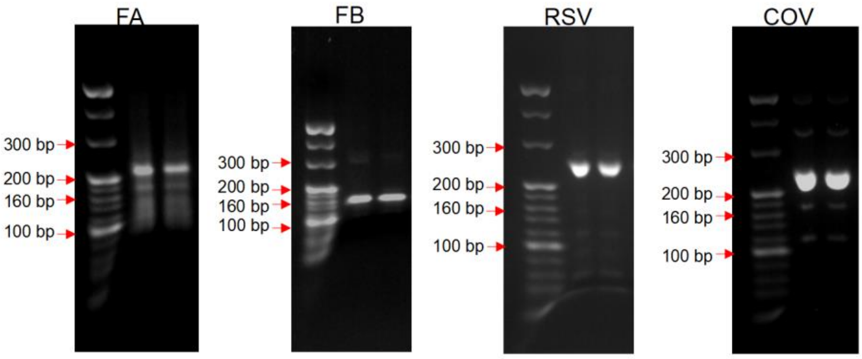

Figure S4. The evaluation of the designed RPA primers of FA, FB, RSV, and COV using RPA amplification at 37 ℃ for 15 min.

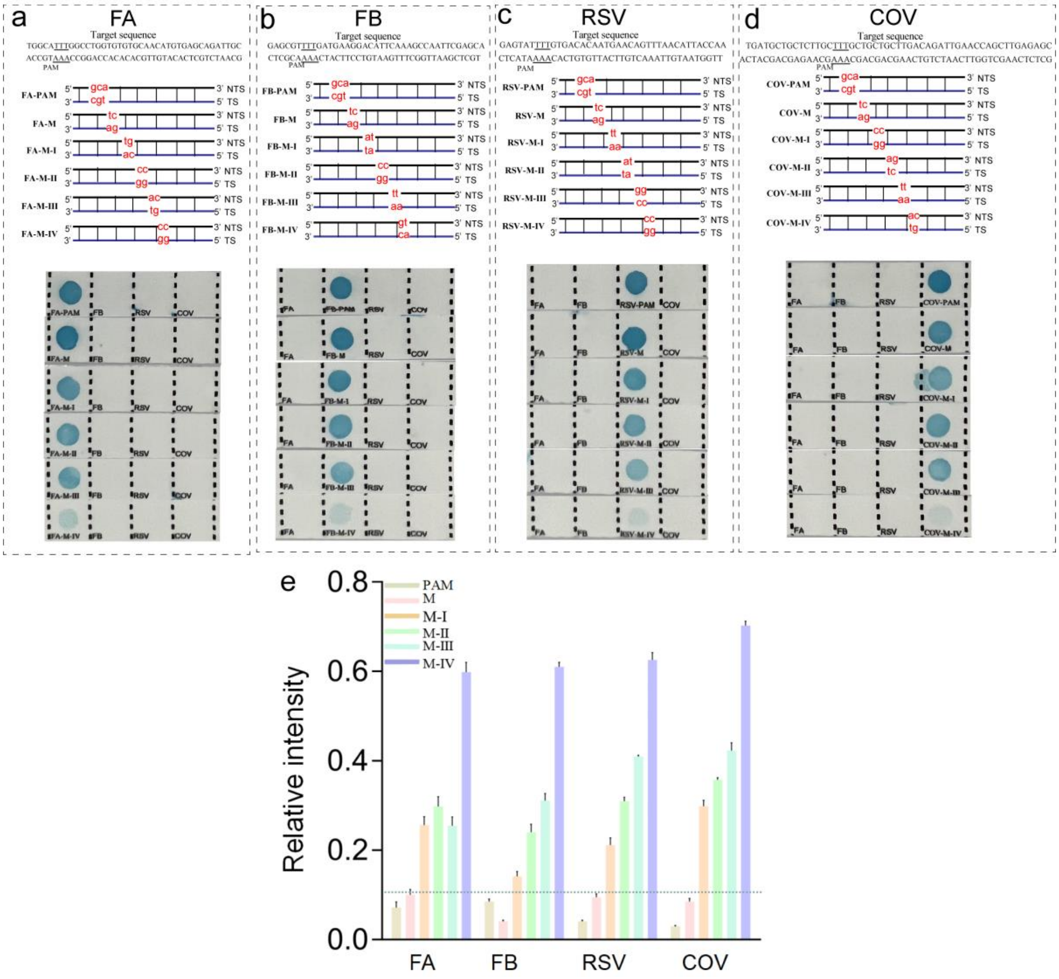

Figure S5. Evaluation of the specificity of the CRISPR-RDB platform using mismatched bases of PAM, M (position 1-2), M-I (position 3-4), M-II (position 5-6), M-III (position 7-8), M-IV (position 9-10) for FA (a), FB (b), RSV (c), and COV (d); Up panel showing target sequences and specific mutations (red font), down panel showing strip results. b) Corresponding color intensity quantitation of strip results (a-d).

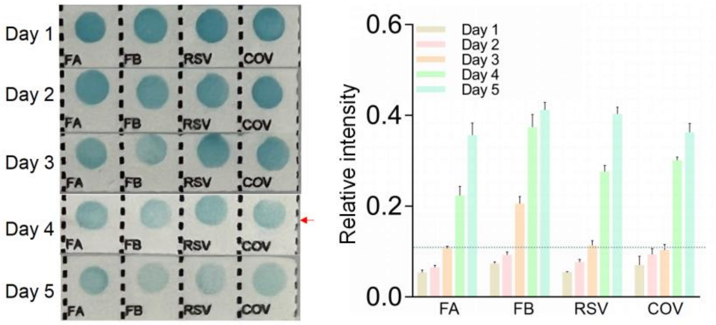

Figure S6. Evaluation of long-term storage of the nylon film probes at 25°C based on visual images (left) and color intensity quantitation (right) from day1 to day 5. Red arrow showing the storage time that this probe will be seriously affected.

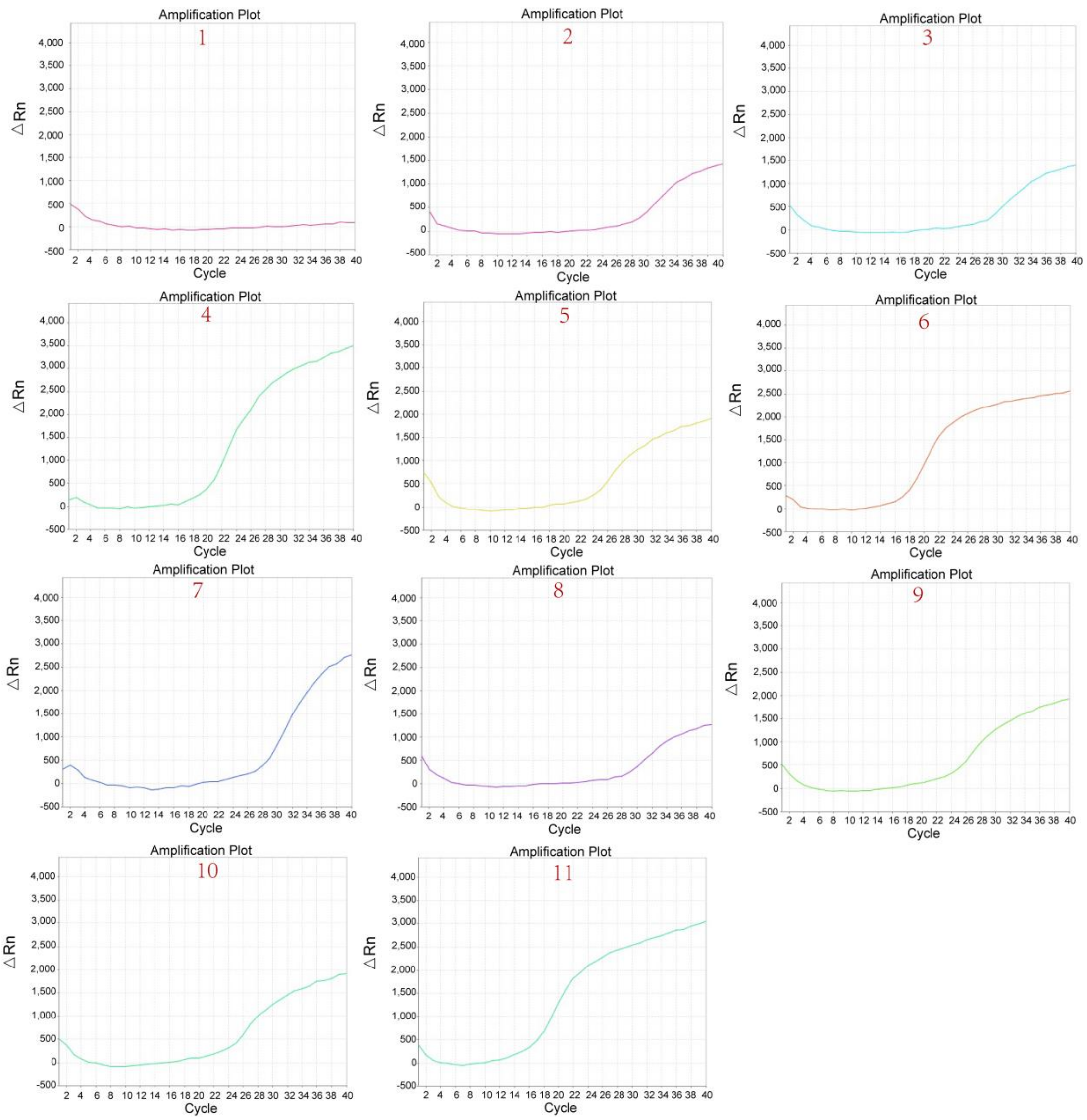

Figure S7. The RT-qPCR detection clinical samples. Negative sample of Nos. 1; FA positive samples of Nos. 2-6; FB positive samples of Nos. 7-11.

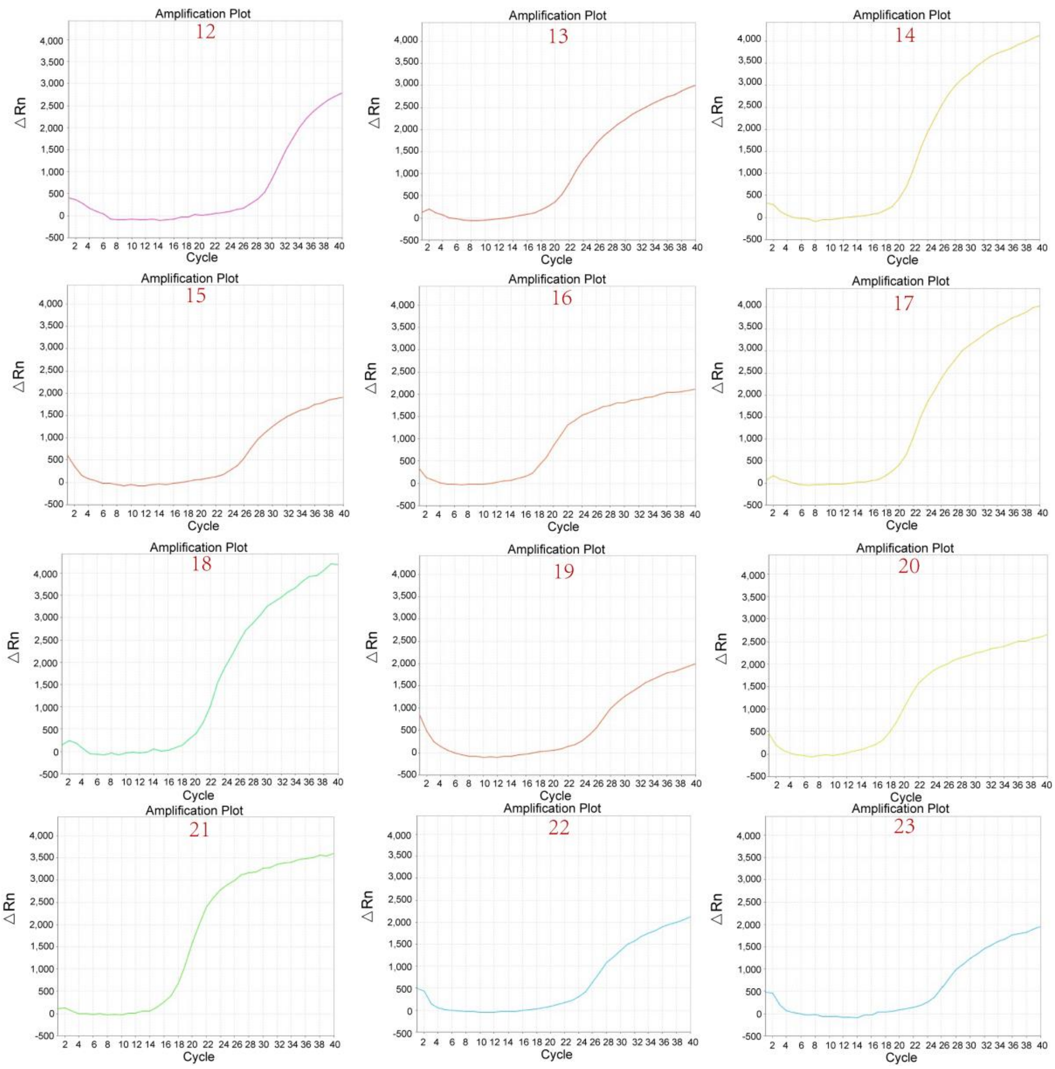

Figure S8. The RT-qPCR detection of clinical samples. RSV positive samples of Nos. 12-16; COV positive samples of Nos. 17-23.

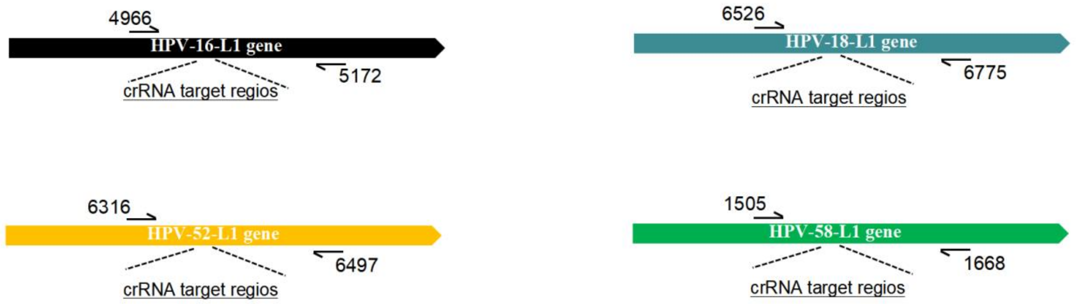

Figure S9. Schematic diagram of the full length of the L1 gene of HPV16/18/52/58 genome and target gene sites for crRNA set and RPA primers sites.

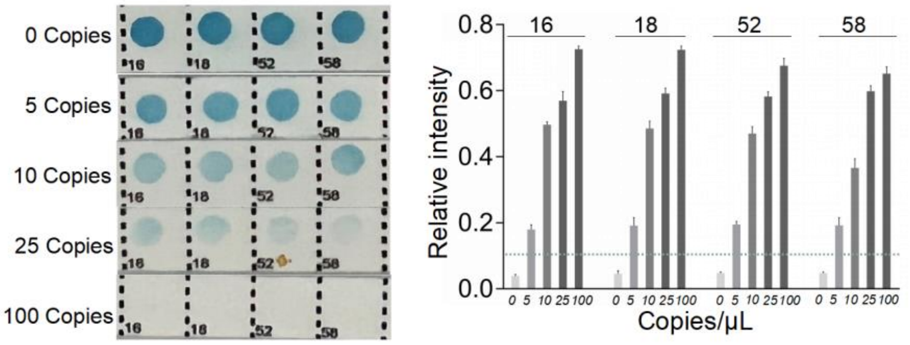

Figure S10. Evaluation of the detection sensitivity for HPV real samples by combining the RPA kit; Visual images (left) and relative intensity quantitation for corresponding strips (left).

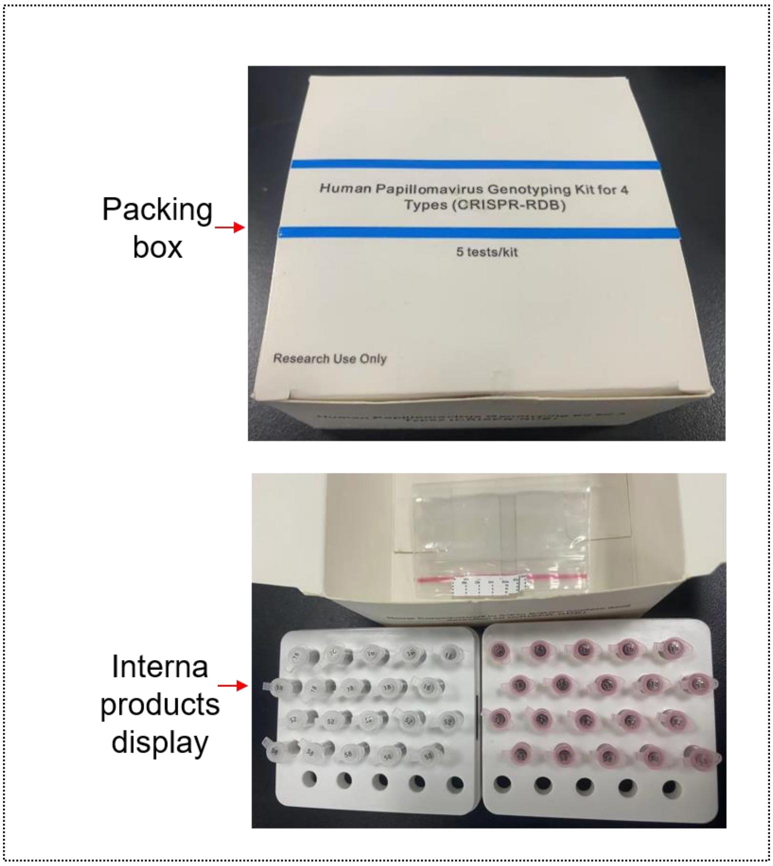

Figure S11. Self-made human papillomavirus genotyping kit for 16/18/52/58 types. White tubes represent the reagent of RPA reaction (16/18/52/58); Red tubes represent the reagent of CRISPR reaction buffer (16/18/52/58); The nylon membrane with for kinds of capDNAs (16/18/52/58).

Table S1. Oligonucleotides used for testing in this study.

Name	Sequence (5’-3’)
CapDNA I	AAAAAATGATGTTCGTTGTG
CapDNA II	AAAAAAAAATTGGTGGACCCTCAGATTCAAC
CapDNA III	AAAAAAAAAACAACGTCGGCCCCAAGGTT
CapDNA IV	AAAAAAAAATTGCCATGTTGAGTGAGAGC
RecDNA I	AAAAAAAAAATTTTTCACAACGAACATCAT
RecDNA II	GTTGAATCTGAGGGTCCACCAA
RecDNA III	AACCTTGGGGCCGACGTTGT
RecDNA IV	GCTCTCACTCAACATGGCAA
FA-crRNA	UAAUUUCUACUAAGUGUAGAUGCCUGGUGUGUGCAACAUGUGAGC
FB-crRNA	UAAUUUCUACUAAGUGUAGAUAUGAAGGACAUUCAAAGCCAAUU
RSV-crRNA	UAAUUUCUACUAAGUGUAGAUUGACACAAUGAACAGUUUAACAUU
COV-crRNA	UAAUUUCUACUAAGUGUAGAUCUGCUGCUUGACAGAUUGAACCA
FA-sense strand	TGGCATTTGGCCTGGTGTGTGCAACATGTGAGCAGATTGC
FA-antisense strand	GCAATCTGCTCACATGTTGCACACACCAGGCCAAATGCCA
FA-PAM-sense strand	TGGCAGCAGGCCTGGTGTGTGCAACATGTGAGCAGATTGC
FA-PAM-antisense strand	GCAATCTGCTCACATGTTGCACACACCAGGCCTGCTGCCA
FA-M-sense strand	TGGCATTTTCCCTGGTGTGTGCAACATGTGAGCAGATTGC
FA-M-antisense strand	GCAATCTGCTCACATGTTGCACACACCAGGGAAAATGCCA
FA-M-I-sense strand	TGGCATTTGGTGTGGTGTGTGCAACATGTGAGCAGATTGC
FA-M-I-antisense strand	GCAATCTGCTCACATGTTGCACACACCACACCAAATGCCA
FA-M-II-sense strand	TGGCATTTGGCCCCGTGTGTGCAACATGTGAGCAGATTGC
FA-M-II-antisense strand	GCAATCTGCTCACATGTTGCACACACGGGGCCAAATGCCA
FA-M-III-sense strand	TGGCATTTGGCCTGACGTGTGCAACATGTGAGCAGATTGC
FA-M-III-antisense strand	GCAATCTGCTCACATGTTGCACACGTCAGGCCAAATGCCA
FA-M-IV-sense strand	TGGCATTTGGCCTGGTCCGTGCAACATGTGAGCAGATTGC
FA-M-IV-antisense strand	GCAATCTGCTCACATGTTGCACGGACCAGGCCAAATGCCA
FB-sense strand	GAGCGTTTTGATGAAGGACATTCAAAGCCAATTCGAGCA
FB-antisense strand	TGCTCGAATTGGCTTTGAATGTCCTTCATCAAAACGCTC
FB-PAM-sense strand	GAGCGTGCAGATGAAGGACATTCAAAGCCAATTCGAGCA
FB-PAM-antisense strand	TGCTCGAATTGGCTTTGAATGTCCTTCATCTGCACGCTC
FB-M-sense strand	GAGCGTTTTTCTGAAGGACATTCAAAGCCAATTCGAGCA
FB-M-antisense strand	TGCTCGAATTGGCTTTGAATGTCCTTCAGAAAAACGCTC
FB-M-I-sense strand	GAGCGTTTTGAATAAGGACATTCAAAGCCAATTCGAGCA
FB-M-I-antisense strand	TGCTCGAATTGGCTTTGAATGTCCTTATTCAAAACGCTC
FB-M-II-sense strand	GAGCGTTTTGATGCCGGACATTCAAAGCCAATTCGAGCA
FB-M-II-antisense strand	TGCTCGAATTGGCTTTGAATGTCCGGCATCAAAACGCTC
FB-M-III-sense strand	GAGCGTTTTGATGAATTACATTCAAAGCCAATTCGAGCA
FB-M-III-antisense strand	TGCTCGAATTGGCTTTGAATGTAATTCATCAAAACGCTC
FB-M-IV-sense strand	GAGCGTTTTGATGAAGGGTATTCAAAGCCAATTCGAGCA
FB-M-IV-antisense strand	TGCTCGAATTGGCTTTGAATACCCTTCATCAAAACGCTC
RSV-sense strand	GAGTATTTTGTGACACAATGAACAGTTTAACATTACCAA

RSV-antisense strand	TTGGTAATGTTAAACTGTTCATTGTGTCACAAAATACTC
RSV-PAM-sense strand	GAGTATGCAGTGACACAATGAACAGTTTAACATTACCAA
RSV-PAM-antisense strand	TTGGTAATGTTAAACTGTTCATTGTGTCACTGCATACTC
RSV-M-sense strand	GAGTATTTTTCGACACAATGAACAGTTTAACATTACCAA
RSV-M-antisense strand	TTGGTAATGTTAAACTGTTCATTGTGTCGAAAAATACTC
RSV-M-I-sense strand	GAGTATTTTGTTTCACAATGAACAGTTTAACATTACCAA
RSV-M-I-antisense strand	TTGGTAATGTTAAACTGTTCATTGTGAAACAAAATACTC
RSV-M-II-sense strand	GAGTATTTTGTGAATCAATGAACAGTTTAACATTACCAA
RSV-M-II-antisense strand	TTGGTAATGTTAAACTGTTCATTGATTCACAAAATACTC
RSV-M-III-sense strand	GAGTATTTTGTGACAGGATGAACAGTTTAACATTACCAA
RSV-M-III-antisense strand	TTGGTAATGTTAAACTGTTCATCCTGTCACAAAATACTC
RSV-M-IV-sense strand	GAGTATTTTGTGACACACCGAACAGTTTAACATTACCAA
RSV-M-IV-antisense strand	TTGGTAATGTTAAACTGTTCGGTGTGTCACAAAATACTC
COV-sense strand	TGATGCTGCTCTTGCTTTGCTGCTGCTTGACAGATTGAACCAGCTTGAGAGC
COV-antisense strand	GCTCTCAAGCTGGTTCAATCTGTCAAGCAGCAGCAAAGCAAGAGCAGCATCA
COV-PAM-sense strand	TGATGCTGCTCTTGCGCAGCTGCTGCTTGACAGATTGAACCAGCTTGAGAGC
COV-PAM-antisense strand	GCTCTCAAGCTGGTTCAATCTGTCAAGCAGCAGCTGCGCAAGAGCAGCATCA
COV-M-sense strand	TGATGCTGCTCTTGCTTTTCTGCTGCTTGACAGATTGAACCAGCTTGAGAGC
COV-M-antisense strand	GCTCTCAAGCTGGTTCAATCTGTCAAGCAGCAGAAAAGCAAGAGCAGCATCA
COV-M-I-sense strand	TGATGCTGCTCTTGCTTTGCCCCTGCTTGACAGATTGAACCAGCTTGAGAGC
COV-M-I-antisense strand	GCTCTCAAGCTGGTTCAATCTGTCAAGCAGGGGCAAAGCAAGAGCAGCATCA
COV-M-II-sense strand	TGATGCTGCTCTTGCTTTGCTGAGGCTTGACAGATTGAACCAGCTTGAGAGC
COV-M-II-antisense strand	GCTCTCAAGCTGGTTCAATCTGTCAAGCCTCAGCAAAGCAAGAGCAGCATCA
COV-M-III-sense strand	TGATGCTGCTCTTGCTTTGCTGCTTTTTGACAGATTGAACCAGCTTGAGAGC
COV-M-III-antisense strand	GCTCTCAAGCTGGTTCAATCTGTCAAAAAGCAGCAAAGCAAGAGCAGCATCA
COV-M-IV-sense strand	TGATGCTGCTCTTGCTTTGCTGCTGCACGACAGATTGAACCAGCTTGAGAGC
COV-M-IV-antisense strand	GCTCTCAAGCTGGTTCAATCTGTCGTGCAGCAGCAAAGCAAGAGCAGCATCA
HPV-16-crRNA	UAAUUUCUACUAAGUGUAGAUGUUUUCCUGACACCUCAUUUUAU
HPV-18-crRNA	UAAUUUCUACUAAGUGUAGAUUUACUGUGGUAGAUACCACUCGCA
HPV-52-crRNA	UAAUUUCUACUAAGUGUAGAUGCAAA UGGCUAGCGA GCCAUAU
HPV-58-crRNA	UAAUUUCUACUAAGUGUAGAUAAAAGUACUAAUUAAAC AGCAC

Table S2. Primers used for isothermal amplification in this study.

Name	Sequence (5'-3')
HPV-16-RPA-FW	AGTATCAGGATTACAATACAGGGTATTTAGA
HPV-16-RPA-RV	CTAGCATTTTCTGTGTCATCCAATTTATTT
HPV-18-RPA-FW	AATGGTGTTTGCTGGCATAATCAATTATTTG
HPV-18-RPA-RV	CATCTGCAGTTAAAGTAATAGTACACAACTG
HPV-52-RPA-FW	TTGATATATGTAGCAGTGTATGTAAGTATCC
HPV-52-RPA-RV	GAGTTAGACCCTTGTATATATAAATCACCTG
HPV-58-RPA-FW	ATGGAGTAAGTTTTATGGAATTAGTTAGACC
HPV-58-RPA-RV	CACGTTAAACATTGTAGGTGTGTATATATAC
FA-RPA-FW	CAACAACCAATCCATTAATAAAACATGAGAAC
FA-RPA-RV	AAATTTTCAAGAAGATCATCCCTTAGACCAG
FA-QPCR-FW	TCCCAGCACAGGTCTCATAG
FA-QPCR-RV	CCTGGCCTGACTAGCAATCT
FB-RPA-FW	GATATACGTAATGTGTTGTCCTTGAGAGTG
FB-RPA-RV	TAAGATCATCAGTAGCAACAAGTTTAGCAA
FB-QPCR-FW	TGTTGTCCTTGAGAGTGTTGG
FB-QPCR-RV	TGAGTTGAGGATCCGATGGC
RSV-RPA-FW	CTTAGCATATGTAGTACAATTACCACTATA
RSV-RPA-RV	TACTTCACTTGGTAATGTTAAACTGTTCAT
RSV-QPCR-FW	TCCCACAAGCTGAAACATGT
RSV-QPCR-RV	ACGGAGCTGCTTACATCTGT
COV-RPA-FW	GAAATTCAACTCCAGGCAGCAGTAGGGGAAC
COV-RPA-RV	GAAAGCTTGTGTTACATTGTATGCTTTAGTG
COV-QPCR-FW	CAACTCCAGGCAGCAGTAGG
COV-QPCR-RV	CAGTACGTTTTTGCCGAGGC

Table S3. Concentrations of clinical samples of FA, FB, RSV, and COV.

**Sample ID***	**Concentration (*10^3 copies/μL)**	**Sample ID**	**Concentration (*10^3 copies/μL)**	**Sample ID**	**Concentration (*10^3 copies/μL)**
1	0.00	9	1.22	17	63.53
2	0.13	10	1.53	18	34.60
3	0.35	11	296.08	19	60.22
4	13.92	12	3.44	20	10.27
5	1.23	13	10.27	21	5.59
6	241.80	14	17.03	22	3.05
7	7.58	15	1.00	23	1.66
8	0.20	16	116.64		

***** NC, sample 1; FA, samples 2-6; FB, samples 7-11; COV, samples 12-16, RSV: samples 17-23.
